# Supplementary material for: Application of GC–TOF/MS and GC×GC–TOF/MS to Discriminate Coffee Products in Three States (Bean, Powder, and Brew)
Source: Foods. 2023 Aug 20;12(16):3123. doi: 10.3390/foods12163123 (PMC10453331; doi:10.3390/foods12163123)
Supplement: Supplementary file 1 [file foods-12-03123-s001.zip › foods-2535360-supplementary.pdf]

Table S1 Identified pyridine, pyrrole, pyrazine, alcohol, phenol, furan, and sulfur-containing compounds in three brands of coffee (CO, PA, ST) under three statuses (bean, powder, and brew) using GC-TOF/MS (μg).

No.	Compounds	RI	Bean			Powder			Brew		
			CO	PA	ST	CO	PA	ST	CO	PA	ST
	**Pyridine (2)**										
1	Pyridine	1170	1.1617±0.1058	2.1207±0.0807	1.1982±0.059	3.7682±0.37	4.1309±0.9219	3.0697±0.3052	0.1028±0.0097	ND	ND
2	3-ethylpyridine	1372	0.0148±0.0014	0.0908±0.001	0.0361±0.0048	0.1751±0.0361	0.1387±0.0325	ND	ND	ND	ND
	**Pyrrole (3)**										
3	1H-pyrrole, 1-(2-furanmethyl)-	1802	0.0537±0.0003	0.1166±0.0028	0.0555±0.0046	0.2398±0.0339	0.2167±0.0526	0.1326±0.0146	0.0164±0.0004	0.0757±0.0232	0.1802±0.1132
4	1H-pyrrole, 1-methyl-	1121	ND	0.2839±0.0016	0.117±0.0288	ND	ND	ND	ND	ND	ND
5	Pyrrole	1495	0.0420±0.0001	0.1319±0.0022	0.0679±0.005	ND	ND	ND	ND	ND	ND
	**Pyrazine (13)**										
6	1,3-diazine	1201	ND	ND	0.1753±0.0113	ND	0.6645±0.1469	ND	ND	ND	ND
7	5-methyl-6,7-dihydro-5H-cyclopentopyrazine	1599	0.0101±0.0014	0.0151±0.0004	0.0078±0.001	ND	ND	ND	ND	ND	ND
8	Pyrazine	1201	0.1668±0.0205	ND	ND	0.7077±0.0718	ND	ND	ND	ND	ND
9	Pyrazine 2-(n-propyl)-	1404	ND	0.0641±0.001	ND	ND	ND	ND	ND	ND	ND
10	2,3-dimethylpyrazine	1336	0.2072±0.0149	0.3843±0.0086	ND	0.6846±0.1369	0.7045±0.1588	0.5802±0.0584	0.008±0.0001	0.0239±0.0078	0.0564±0.0391
11	2,5-dimethylpyrazine	1311	0.5754±0.0225	1.2958±0.0562	0.4587±0.2101	1.9477±0.8659	1.0877±0.2411	2.7337±0.3003	0.0364±0.0006	0.0447±0.0288	0.1549±0.1081
12	Pyrazine, 2-vinyl-6-methyl-	1477	ND	ND	ND	0.0474±0.00071	0.0732±0.0172	0.0414±0.0052	0.0185±0.0003	ND	0.0887±0.0571
13	Pyrazine 2-ethyl-5-methyl-	1379	ND	0.3042±0.0026	0.1819±0.0188	0.585±0.11706	0.7157±0.1674	0.695±0.0773	0.0181±0.0004	0.049±0.0148	0.0992±0.0655
14	Pyrazine, 2-ethyl-6-methyl-	1373	0.2543±0.0256	0.3509±0.0046	0.2055±0.0189	0.6619±0.1335	0.7842±0.181	0.7452±0.0793	0.007±0.0001	0.0185±0.0056	0.0363±0.0234
15	Pyrazine, 3-ethyl-2,5-dimethyl-	1437	0.0947±0.0245	ND	ND	ND	0.1489±0.0355	0.1226±0.0141	ND	ND	ND
16	Pyrazine, vinyl-	1426	0.0119±0.0005	0.0292±0.0012	0.0156±0.0023	ND	ND	ND	ND	ND	ND
17	Pyrazine, methyl-	1255	1.0593±0.0773	1.2848±0.0458	ND	2.3459±0.3288	2.7727±0.626	2.3864±0.222	0.0914±0.0037	0.2237±0.0696	0.4749±0.3252
18	Pyrazine trimethyl	1394	ND	0.4037±0.0025	ND	0.7661±0.0693	ND	0.8265±0.0971	ND	ND	0.0729±0.05
	**Alcohols (16)**										
19	1,6-heptadien-4-ol	1428	ND	0.0435±0.0005	0.0242±0.0054	ND	ND	ND	ND	ND	ND
20	(S)-(-)-1,2,4-butanetriol	1429	ND	ND	ND	ND	ND	ND	0.0006±0	0.002±0.0008	ND
21	2-butanol, 3-methyl-	1480	ND	0.0459±0.0094	0.0198±0.0073	0.0429±0.023	0.0776±0.0175	0.1992±0.1549	ND	0.0005±0.0001	ND
22	2-propyl-1-pentanol	1207	ND	0.0632±0.0146	0.0296±0.0063	0.0928±0.0079	ND	0.0711±0.0077	0.0039±0.0007	ND	0.0023±0.0014

23	2-thiophene ethanol	1905	0.0023±0.00001	0.004±0.0001	0.0024±0.0002	ND	0.0095±0.0023	0.0065±0.0007	0.0002±0	0.0008±0.0002	0.0016±0.001
24	4-pyridinemethanol	1960	ND	ND	ND	ND	ND	ND	ND	0.0009±0.0003	ND
25	3-butan-1-ol, 3-methyl-	1242	0.0277±0.00039	0.0392±0.0002	0.0214±0.0008	0.0549±0.00056	0.0616±0.012	0.0559±0.0077	ND	ND	ND
26	3-pyridinol	2333	ND	0.0007±0	ND	0.0049±0.00012	0.0022±0.0006	ND	ND	ND	ND
27	Benzyl alcohol	1850	ND	ND	ND	0.0039±0.00008	0.004±0.0008	0.0031±0.0003	0.0004±0	0.0003±0.0001	0.0007±0.0005
28	Linalool	1541	ND	ND	ND	ND	ND	ND	0.001±0	0.0018±0.0006	0.0039±0.0025
29	Tetraethylene glycol	2712	ND	ND	ND	ND	ND	ND	ND	ND	0.0011±0.0008
30	Crotyl alcohol	1708	ND	ND	0.008±0.0014	ND	ND	ND	ND	ND	ND
31	Cyclopropylmethanol	1952	ND	0.0581±0.0076	0.0085±0.0021	0.1259±0.0238	0.0571±0.0161	0.0134±0.0022	ND	ND	ND
32	Ethanol 2,2'-oxydi-	1271	0.2951±0.0355	ND	ND	0.5609±0.0609	ND	0.7879±0.0868	ND	ND	ND
33	Furanol	1936	0.0065±0.00002	0.0082±0.0017	ND	ND	ND	ND	ND	ND	ND
34	Methyl mercaptan	909	ND	0.0007±0	0.0002±0	ND	ND	ND	ND	ND	ND
	**Phenols (12)**										
35	2-methoxy-4-vinylphenol	1988	0.0074±0.00009	0.0139±0.0013	0.0056±0.0005	0.0264±0.00048	0.0239±0.007	0.0181±0.0022	0.001±0.0001	0.0029±0.0007	0.0045±0.0024
36	Maltol	1913	0.0149±0.00003	0.0591±0.0055	0.0264±0.0044	0.1252±0.0248	0.1607±0.059	0.084±0.0139	0.0023±0.0001	0.0067±0.0019	0.0018±0.0011
37	P-cresol	1952	ND	ND	ND	ND	ND	ND	0.0003±0	0.0014±0.0004	ND
38	phenol	1926	0.0144±0.00021	0.0784±0.0024	0.0307±0.0026	0.1536±0.032	0.1177±0.0285	0.04±0.0044	0.004±0.0001	0.028±0.009	0.0929±0.0641
39	2,3-dimethylphenol	1974	ND	ND	ND	ND	ND	ND	ND	0.0003±0.0001	0.0434±0.0377
40	Phenol 2-methoxy	1833	0.0224±0.00017	0.1133±0.0068	0.0452±0.0047	0.2188±0.0323	0.1611±0.0394	0.0569±0.0065	0.0031±0.0001	0.0209±0.0067	0.0637±0.0408
41	3,4-dimethylphenol	1996	ND	ND	ND	ND	ND	ND	ND	0.0001±0	0.0003±0.0002
42	2-methylphenol	1926	ND	0.0093±0.0003	0.0043±0.0004	0.0174±0.00027	0.0158±0.0038	0.003±0.0029	ND	ND	ND
43	3-methylphenol	1140	ND	ND	ND	0.2875±0.0233	0.2927±0.0606	0.221±0.0216	0.0089±0.0025	0.0553±0.0315	0.0052±0.0035
44	4-ethylphenol	1982	0.0003±0	0.0014±0.0001	0.0004±0	0.0026±0.0004	ND	0.0008±0.0001	0.0002±0	0.0009±0.0003	0.0026±0.0017
45	Phenol 4-ethyl-2-methoxy	1935	ND	0.0182±0.0013	0.0058±0.0005	0.0341±0.00062	0.0221±0.006	0.0106±0.0013	0.0008±0	0.0063±0.002	0.0211±0.0136
46	P-tert-butylcatechol	2407	ND	ND	ND	ND	0.0001±0	ND	ND	ND	ND
	**Furan (17)**										
47	2,2'-bisfuran	1575	ND	ND	ND	ND	ND	ND	0.0044±0.0003	0.0155±0.0053	0.0284±0.0181
48	2,4-dimethylfuran	1001	ND	ND	ND	ND	ND	ND	ND	0.0049±0.0017	ND
49	2-vinyl furan	1053	ND	ND	ND	ND	ND	ND	ND	0.0535±0.0219	0.1118±0.0775
50	2,3-dihydrobenzofuran	2262	ND	0.0004±0	0.0002±0	0.0008±0.00001	0.0008±0.0002	0.0007±0.0001	0.0014±0.0004	0.0003±0.0001	0.0006±0.0004
51	Furan	919	0.006±0.0005	0.0235±0.0063	0.0054±0.002	0.0372±0.0004	0.0339±0.0074	0.0255±0.0046	0.0035±0.0002	0.0161±0.0057	0.0378±0.0278

52	Furan 2-(2-furylmethyl)-5-methyl-	1660	0.0116±0.0006	0.045±0.0009	0.0199±0.002	0.0909±0.0177	0.0772±0.0184	0.031±0.0034	0.0037±0.0001	0.0295±0.0096	0.0803±0.0515
53	Furan 2-(2-propenyl)-	1071	ND	ND	ND	ND	ND	ND	ND	0.0027±0.001	0.0065±0.0048
54	Furan 2-(methoxymethyl)-	1231	0.0569±0.0054	0.1887±0.0044	0.1085±0.0116	0.2801±0.0233	0.3437±0.0719	0.1924±0.0202	0.0163±0.001	0.0978±0.0271	0.1369±0.0586
55	Furan 2,2'-[oxybis(methylene)] bis-	1920	0.0054±0.0009	0.0224±0.0013	0.0092±0.0008	0.0447±0.0066	0.0357±0.0092	0.0137±0.0015	0.0014±0	0.01±0.0031	0.0286±0.0184
56	Furan, 2,2'-methylenebis-	1590	0.0417±0.0024	0.1792±0.0015	0.0764±0.0084	0.3509±0.068	0.2913±0.0717	0.1269±0.0148	ND	ND	ND
57	2,5-dimethylfuran	963	0.0133±0.0006	0.0926±0.0116	0.0384±0.0061	0.1107±0.0068	0.1271±0.0254	0.0565±0.0064	0.0077±0.0032	0.0817±0.0247	0.1579±0.1054
58	Furan 2-[[(methylthio)methyl]-	1469	0.0422±0.0021	0.2997±0.0248	0.1452±0.0222	0.6168±0.1241	0.6167±0.139	0.2137±0.0274	0.0088±0.0005	0.1005±0.0221	0.1004±0.0616
59	Furan, 2-ethyl-	980	0.0021±0.0001	0.0161±0.0004	0.006±0.001	0.0132±0.0008	0.0147±0.0028	0.0071±0.0009	ND	ND	ND
60	Furan, 2-ethyl-5-methyl-	1488	ND	ND	ND	1.1139±0.1464	0.0124±0.0026	0.0111±0.0069	ND	ND	ND
61	Furan, 2-methyl-	933	0.0379±0.0008	0.1075±0.0096	0.0738±0.0154	0.2092±0.0167	0.1335±0.027	0.1163±0.0157	0.0166±0.001	0.1044±0.0358	0.2538±0.1852
62	Furan, 2-pentyl	1219	ND	ND	ND	ND	ND	ND	0.0035±0.0006	0.0119±0.0085	ND
63	Furan, 3-phenyl	1828	ND	ND	ND	ND	ND	ND	0.0007±0	0.0024±0.0008	0.0056±0.0033
						**Sulfur-containing compounds (13)**				
64	Dihydro-3-(2H)-thiophene	1538	0.011±0.0003	0.0156±0.0017	0.0077±0.0011	ND	ND	ND	ND	ND	ND
65	Dimethyl sulfide	914	0.0031±0.0007	0.0054±0.0005	0.0015±0.0008	ND	ND	ND	ND	ND	ND
66	Dodecyl sulfate	1138	ND	ND	0.0106±0.0052	ND	ND	ND	ND	ND	ND
67	Thiophene, 2-methoxy-5-methyl-	1367	ND	ND	0.0062±0.0035	ND	ND	ND	ND	ND	ND
68	Thiazole	1239	ND	ND	ND	0.026±0.0036	ND	ND	ND	ND	ND
69	Thiazole 2-methyl-	1229	ND	ND	ND	0.009±0.0015	ND	ND	ND	ND	ND
70	Thiophene, 2-methoxy-5-methyl-	1367	ND	ND	ND	ND	ND	0.008±0.003	ND	ND	ND
71	Carbon disulfide	1720	ND	ND	ND	ND	ND	ND	0.0025±0.0004	0.0058±0.0013	0.0121±0.0082
72	Dimethyl disulfide	1048	ND	ND	ND	ND	ND	ND	0.016±0.0094	0.0576±0.0229	ND
73	Thiophene	1003	ND	ND	ND	ND	ND	ND	ND	0.0228±0.0072	0.0503±0.0357
74	Thiophene 3-methyl	1092	ND	ND	ND	ND	ND	ND	ND	0.0108±0.0033	0.0224±0.0164
75	Thiophene 2-methyl	1069	ND	0.0704±0.0012	ND	0.0705±0.0059	0.0622±0.0123	0.0328±0.0033	0.0066±0.0013	0.0441±0.0196	0.1045±0.0752
76	Phenothiamine	1530	0.0027±0.0007	ND	ND	ND	ND	ND	ND	ND	ND

Table S2. Identified pyridine, pyrrole, pyrazine, alcohol, phenol, furan, and sulfur-containing compounds in three brands of coffee (CO, PA, ST) under three statuses (bean, powder, and brew) using GC×GC-TOF/MS (μg).

No.	Compounds	RI	Bean			Powder			Brew		
			CO	PA	ST	CO	PA	ST	CO	PA	ST
	**Pyridine (12)**										
1	dimethyl pyridine	1218	0.0023±0.0008	0.0041±0.0016	ND	ND	0.0167±0.0047	ND	ND	ND	ND
2	Pyridine	1123	1.1309±0.1219	1.5036±0.1038	1.1627±0.1059	3.2992±0.0719	4.669±0.0185	3.373±0.0045	0.6374±0.0122	0.121±0.0009	0.206±0.0038
3	Pyridine 2,3-dimethyl	1349	0.0004±0.0002	ND	0.0009±0.0002	ND	ND	ND	ND	ND	ND
4	Pyridine, 1-acetyl-1,2,3,4-tetrahydro-	1795	ND	ND	ND	ND	0.0017±0.0004	0.0009±0	ND	ND	ND
5	Pyridine 2-ethyl-	1251	0.0032±0.001	0.0095±0.0009	ND	0.0116±0.0016	0.0386±0.0036	0.0313±0.0022	*	0.0012±0.0001	0.0012±0.0015
6	Pyridine 2-ethyl-6-methyl-	1274	ND	ND	ND	0.0026±0.0005	0.0093±0.001	0.0072±0.0006	ND	ND	ND
7	Pyridine 2-methyl	1177	0.0165±0.0075	0.0379±0.006	0.0144±0.0004	0.0654±0.0163	0.1704±0.0193	ND	ND	0.0038±0.0005	ND
8	Pyridine, 3-vinyl	1466	0.0008±0.0002	0.0017±0.0004	0.00063±0.0001	0.0029±0.0004	0.0079±0.0009	0.0059±0.0004	ND	ND	0.0005±0.0006
9	3-ethylpyridine	1364	0.0155±0.0042	0.0482±0.0086	ND	0.0561±0.0065	ND	ND	0.0015±0.0004	0.0111±0.0011	0.0132±0.0016
10	3-methylpyridine	1272	0.0113±0.0018	0.0346±0.002	ND	0.046±0.0082	0.1651±0.0138	ND	ND	0.0055±0.0003	ND
11	Pyridine 3-propyl	1455	ND	ND	ND	ND	0.0082±0.0008	0.009±0.0011	ND	ND	ND
12	Pyridine, 4-ethyl-	1381	ND	0.0005±0	ND	ND	0.002±0.0003	ND	ND	ND	ND
	**Pyrrole (8)**										
13	1H-pyrrole, 1-(2-furanmethyl)-	1806	0.0808±0.0166	ND	ND	0.2174±0.0228	0.4749±0.0407	0.4000±0.000	0.0621±0.0127	0.1273±0.0158	0.1033±0.0046
14	1H-pyrrole, 1-butyl-	1216	ND	ND	ND	ND	ND	ND	0.0034±0.0006	0.013±0.0011	0.0167±0.0014
15	1H-pyrrole, 1-ethyl-	1118	0.0096±0.0041	0.0149±0.0002	ND	0.0347±0.0049	0.0744±0.0066	ND	0.0104±0.0021	0.0318±0.0013	0.0481±0.0052
16	1H-pyrrole, 1-methyl-	1054	0.0463±0.0212	0.1264±0.0159	ND	0.1712±0.0396	0.4473±0.0419	ND	0.0563±0.0148	0.097±0.0058	ND
17	1H-pyrrole, 2,4-dimethyl-	1579	ND	0.0028±0.0006	0.0012±0.0004	0.006±0.0005	0.0996±0.0101	0.0804±0.0058	ND	ND	0.0500±0.0003
18	1H-pyrrole, 2-ethyl-4-methyl-	1645	ND	ND	ND	0.0106±0.0016	0.043±0.004	0.0349±0.0025	ND	ND	ND
19	1H-pyrrole, 3-methyl-	1531	ND	ND	ND	0.0040±0.0001	0.1484±0.0108	0.0082±0.001	ND	ND	ND
20	Pyrrole	1488	0.0463±0.0147	0.1084±0.0098	0.0369±0.0023	0.1795±0.0182	0.481±0.0456	0.4209±0.0381	0.0201±0.0057	0.0272±0.0013	0.0152±0.0081
	**Pyrazine (36)**										
21	2,3-diethylpyrazine	1436	0.003±0.0008	0.0033±0.0007	ND	0.0089±0.0011	0.0133±0.0012	0.0071±0.0012	0.0008±0.0003	ND	0.0005±0.001
22	2,3-dimethyl-5-ethylpyrazine	1445	0.0255±0.0074	0.0415±0.0032	ND	0.0764±0.0093	0.1011±0.0034	0.0781±0.0092	0.0079±0.002	0.0122±0.0019	0.0064±0.005
23	2-acetyl-3-methylpyrazine	1681	0.0149±0.0043	0.0253±0.0037	ND	0.0448±0.0061	0.0567±0.0034	0.0342±0.0027	0.001±0.004	0.0058±0.0006	0.0001±0.002
24	2-butyl-3-methylpyrazine	1617	0.0007±0.0002	0.0009±0.0001	0.0002±0.002	0.0025±0.0005	0.0034±0.0003	0.0025±0.0004	ND	ND	ND
25	2-isopentyl-6-methylpyrazine	1610	0.0012±0.0003	0.0019±0.0001	ND	0.0051±0.0008	0.0061±0.0007	0.005±0.009	ND	0.0011±0.0002	0.0006±0.006

26	2-isoamylpyrazine	1566	0.0006±0.0002	0.0008±0.0001	0.0003±0.0001	0.0021±0.0004	ND	ND	ND	ND	ND
27	2-isobutyl-3-methylpyrazine	1472	0.0019±0.0006	0.0028±0.0003	0.0048±0.0006	0.0059±0.0008	ND	0.0059±0.0005	ND	ND	ND
28	2-isopropylpyrazine	1326	0.0017±0.0005	ND	0.0098±0.0009	0.0062±0.0007	ND	ND	ND	0.0006±0.0001	0.0004±0.0002
29	2-methyl-3-propylpyrazine	1462	ND	ND	0.0019±0.0001	ND	ND	0.0181±0.001	ND	0.0019±0.0003	0.001±0.0007
30	3H, 4H-pyrrolo[1,2-a]pyrazine	1629	0.011±0.0014	0.0205±0.0011	ND	0.0308±0.0041	0.0719±0.0058	0.0567±0.0051	0.0007±0.0002	0.002±0.0002	0.0019±0.0001
31	4-methylpyrrolo[1,2-a]pyrazine	1986	ND	0.0003±0	ND	ND	0.0012±0.0002	0.0006±0.0001	ND	ND	ND
32	Acetylpyrazine	1611	0.0226±0.0084	0.015±0.0023	ND	ND	0.0572±0.0183	ND	ND	ND	ND
33	Pyrazine	1159	0.2614±0.1021	0.303±0.0658	0.1056±0.0699	0.7738±0.2447	0.9577±0.0957	0.762±0.0649	0.0822±0.0175	0.1034±0.0075	0.0953±0.0026
34	Pyrazine, (1-methylvinyl)-	1583	0.0078±0.002	0.0128±0.0009	0.0121±0.0004	0.0134±0.0018	0.0312±0.0022	0.0294±0.0018	ND	ND	ND
35	Pyrazine, (2-methylpropyl)-	1429	0.0014±0.0004	0.0021±0.0002	0.0008±0.0001	0.0043±0.0005	0.0082±0.0008	ND	ND	ND	ND
36	Pyrazine 2-(n-propyl)-	1397	0.0178±0.0043	0.0336±0.0026	ND	0.0576±0.0063	0.132±0.0102	0.1055±0.0075	ND	ND	0.0645±0.0036
37	2,3-dimethylpyrazine	1323	0.341±0.0688	0.2359±0.0207	ND	0.5679±0.0546	ND	0.7576±0.165	ND	0.0524±0.0053	ND
38	Pyrazine, 2,5-diethyl-	1440	ND	0.0044±0.0006	ND	ND	0.0156±0.0059	ND	ND	0.0014±0.0002	ND
39	2,5-dimethylpyrazine	1297	0.9326±0.1033	0.9367±0.1396	ND	ND	ND	0.0027±0.001	0.0833±0.0088	0.1147±0.0202	ND
40	Pyrazine, 2,6-diethyl-	1416	0.0176±0.005	0.0215±0.0018	ND	0.0628±0.0072	0.0911±0.0079	0.0512±0.0039	0.0044±0.0011	0.0074±0.0012	0.0036±0.0002
41	2,6-dimethylpyrazine	1302	0.5018±0.142	ND	ND	ND	ND	0.288±0.0713	0.2694±0.0945	ND	0.1680±0.0394
42	Pyrazine, 2-butyl-3,5-dimethyl-	1649	ND	ND	0.0174±0.0015	0.054±0.0058	0.0936±0.007	0.0451±0.0053	ND	ND	ND
43	Pyrazine 2-ethyl-5-methyl-	1369	0.0536±0.0113	0.0535±0.0089	ND	0.0054±0.0005	0.1876±0.0156	ND	0.0438±0.0093	0.0416±0.0062	0.0309±0.0025
44	Pyrazine, 2-ethyl-6-methyl-	1363	0.0625±0.0051	0.0869±0.0151	ND	0.3136±0.1049	ND	0.2444±0.081	0.0494±0.0198	0.0784±0.0303	0.0502±0.0067
45	Pyrazine 2-methoxy-3-(2-methylpropyl)-	1508	0.003±0.0005	0.0038±0.0003	ND	0.0092±0.0011	0.0141±0.0012	0.0079±0.0007	0.0023±0.0005	0.0033±0.0003	0.0018±0.0006
46	Pyrazine, 2-methyl-6-(1-propenyl)-, (E)-	1643	ND	ND	ND	ND	0.0076±0.0006	ND	ND	ND	ND
47	Pyrazine, 2-methyl-5-(1-propenyl)-, (Z)-	1649	0.0008±0.0002	0.0009±0.0001	ND	ND	ND	ND	ND	ND	ND
48	Pyrazine, 2-methyl-5-propyl-	1460	0.004±0.0012	0.0005±0.0001	ND	0.0233±0.003	0.032±0.0029	0.0198±0.0018	ND	ND	ND
49	Pyrazine 3,5-diethyl-2-methyl-	1479	0.0076±0.0023	0.008±0.0015	ND	ND	ND	0.0005±0.0001	ND	ND	ND
50	Pyrazine, 3-ethyl-2,5-dimethyl-	1428	ND	0.0318±0.0029	ND	0.1187±0.0675	0.1137±0.006	0.0682±0.0076	0.02±0.0042	0.0299±0.0048	0.0146±0.0015
51	Pyrazine, vinyl-	1419	0.017±0.0047	0.0237±0.0016	ND	0.0601±0.008	0.1002±0.0079	0.0559±0.003	0.0026±0.0006	0.0047±0.0004	0.0027±0.0002
52	Pyrazine, ethyl-	1306	ND	0.1466±0.0074	ND	0.0049±0.0017	ND	0.3892±0.0515	ND	ND	ND
53	Pyrazine, methyl-	1228	0.4215±0.0963	0.9261±0.116	ND	0.7254±0.0698	0.0015±0.0001	0.3209±0.0324	0.3991±0.0828	0.4326±0.0347	ND
54	Pyrazine tetramethyl	1462	0.0030±0.0008	0.0042±0.0004	0.0012±0.0001	0.0089±0.0012	ND	0.0105±0.0004	ND	ND	ND

55	Pyrazine trimethyl	1399	ND	0.0019±0.0002	ND	ND	0.358±0.0183	0.0014±0.0004	ND	ND	ND
56	Pyrrolo[1,2-a]pyrazine	1982	ND	0.0005±0	ND	ND	ND	0.0015±0.0001	ND	ND	ND
	**Alcohols (66)**										
57	1,2-butanediol	1662	ND	0.0006±0.0001	ND	ND	ND	ND	ND	ND	ND
58	E)-1,3-butadien-1-ol	1228	ND	ND	ND	0.0043±0.0004	ND	ND	ND	ND	ND
59	1,3-propanediol	1569	0.0026±0.0005	0.0032±0.0003	0.00108±0.0004	0.006±0.005	0.0092±0.0013	0.0059±0.0005	ND	ND	ND
60	1,4-benzenediol, 2,6-dimethyl-	2866	ND	ND	ND	ND	ND	ND	ND	ND	0.0003±0.0008
61	1,6-heptadien-4-ol	1519	ND	ND	ND	0.0017±0.0003	0.0303±0.0022	0.0309±0.0055	ND	ND	0.0022±0.0006
62	1-butanol	1057	ND	0.0009±0	ND	ND	ND	ND	0.0006±0.0002	0.0005±0.0001	0.0006±0.0002
63	1-butanol, 3-methyl-	1150	ND	ND	0.0042±0.0001	0.029±0.0039	0.0503±0.0042	ND	0.0086±0.0022	ND	ND
64	1-hexanol	1326	0.0115±0.0022	0.0093±0.0022	ND	0.039±0.0042	0.0372±0.0036	0.031±0.0026	0.0054±0.0012	0.0054±0.0004	0.0034±0.0005
65	1-octyl-3-ol	1431	0.0085±0.0012	0.0122±0.0008	0.0322±0.0086	0.0251±0.0029	0.0463±0.0048	0.0274±0.0021	0.0054±0.0013	0.008±0.0009	0.0044±0.0001
66	1-pentanol	1208	0.0008±0.0001	0.0008±0.0001	0.0026±0.0003	0.0024±0.0002	0.0029±0.0006	0.0021±0.0002	ND	0.0004±0	ND
67	1-penten-3-ol	1318	0.0075±0.0021	0.0136±0.0014	ND	ND	ND	ND	ND	ND	ND
68	1-propanol	819	ND	ND	ND	ND	ND	0.0011±0	ND	ND	ND
69	1-propanol, 2-methyl-	957	0.0016±0.0006	0.0027±0.0002	0.0086±0.0002	0.0053±0.0016	0.0106±0.0011	0.0059±0.0004	ND	ND	ND
70	2,3-butanediol	1519	ND	0.1114±0.0098	0.0017±0.0005	ND	0.2736±0.0285	ND	ND	ND	ND
71	2,3-Butanediol, [S-(R*, R*)]-	1557	0.1357±0.0289	0.1592±0.0202	ND	0.3644±0.0283	0.4557±0.0335	ND	ND	ND	ND
72	2-butan-1-ol, 3-methyl-acetic acid	1214	0.0088±0.0038	ND	0.0268±0.0014	0.0052±0.0006	0.0472±0.005	0.0321±0.0016	0.0080±0.0011	0.0122±0.001	0.0070±0.0002
73	2-furan methanol	1637	0.5022±0.1857	ND	ND	ND	ND	ND	0.4542±0.0713	0.2792±0.0518	0.2274±0.0317
74	2-furanmethanol, 5-vinyltetrahydro-β5-trimethyl-, cis-	1424	ND	ND	ND	ND	ND	ND	0.0052±0.0012	ND	0.0042±0.0005
75	2-furanmethanol, 5-methyl-	1700	0.0033±0.0006	0.002±0.0001	ND	0.0088±0.001	0.0068±0.0006	0.0034±0.0003	0.0004±0.0001	0.0009±0.0003	ND
76	2-furan methanol, acetate	1516	0.2117±0.0396	ND	0.0091±0.0023	0.5219±0.0562	0.1594±0.0433	0.1634±0.0087	0.0019±0.0005	ND	0.0013±0.0003
77	2-furanol, tetrahydro-	1463	ND	ND	ND	0.003±0.0003	0.01±0.0037	0.0012±0.0001	ND	ND	ND
78	2-furanol, tetrahydro-2-methyl-	1476	ND	0.0003±0	0.0191±0.0032	ND	ND	ND	ND	ND	ND
79	2-furfuryl mercaptan	1407	0.003±0.0006	0.0026±0.0029	0.0005±0.0001	0.013±0.0006	0.0126±0.0029	0.0015±0.0001	ND	ND	ND
80	2-heptanol (S)-	1291	0.0104±0.0015	0.0192±0.0018	ND	0.0292±0.0022	0.0741±0.0069	0.0557±0.0101	ND	0.0165±0.0013	0.0099±0.0002
81	2-Hexanol (S)-	1169	ND	ND	0.0003±0.0001	ND	0.0044±0.0002	ND	ND	0.001±0.0001	ND
82	2-hex-1-ol	1663	ND	0.0005±0.0003	ND	ND	0.0017±0.0006	ND	ND	ND	ND

83	2H-pyran-2-methanol, tetrahydro-	1458	0.0006±0.0001	0.0023±0.0002	0.0007±0.0001	0.0017±0.0002	0.0088±0.0009	0.0062±0.0005	ND	ND	ND
84	2-methyl-5-hexene-3-ol	1222	0.0011±0.0003	0.0008±0.0001	ND	0.0098±0.0012	ND	ND	ND	ND	ND
85	2-propanol 1-(2-methoxy-1-methylethoxy)-	1458	0.0011±0.0002	ND	ND	ND	0.0028±0.0004	0.0152±0.0035	ND	ND	ND
86	2-propanol, 1-methoxy	1033	ND	0.001±0.0002	ND	0.0036±0.0008	0.0121±0.0013	0.0089±0.001	ND	ND	ND
87	2-propan-1-ol	1006	0.0005±0.0002	0.0014±0.0001	0.0003±0.0001	0.0031±0.0004	0.0191±0.0016	ND	ND	ND	ND
88	2-propanol, 2-methyl-	1135	ND	ND	ND	ND	ND	ND	0.0016±0.0003	0.0017±0.0001	ND
89	2-thiophene ethanol	1916	0.0055±0.001	0.0088±0.0005	ND	ND	ND	ND	ND	ND	ND
90	3-butan-2-ol, 2-methyl-	1632	ND	ND	ND	0.0301±0.0085	0.0282±0.0037	0.0121±0.0006	0.0071±0.0027	0.0081±0.0004	0.0056±0.0003
91	3-butan-1-ol, 3-methyl-	1204	ND	0.0213±0.0016	0.0062±0.0005	ND	ND	ND	0.0019±0.0003	0.0012±0	ND
92	3-mercapto-3-methylbutanol	1641	0.0049±0.0008	0.0105±0.0027	ND	0.0403±0.0017	ND	0.0608±0.0036	ND	ND	ND
93	3-methyl-2-pyrazinyl methanol	1890	ND	0.0003±0	ND	0.0013±0.0004	0.0009±0.0001	ND	ND	ND	ND
94	3-methyl-3-butene-1-thiol	665	ND	0.0007±0.0001	0.0004±0.0001	ND	0.0022±0.0002	ND	ND	ND	ND
95	3-octanol	1373	ND	0.0025±0.0002	ND	0.0033±0.0004	0.0096±0.001	0.0049±0.0006	ND	0.0012±0.0001	0.0057±0.0005
96	3-thiophene ethanol	1916	ND	ND	ND	ND	ND	ND	ND	0.0013±0.0001	ND
97	4-penten-1-ol	1081	ND	0.0091±0.0026	ND	ND	ND	ND	ND	0.0148±0.0007	ND
98	3-pentanol	998	ND	ND	ND	ND	0.0039±0.0005	0.0023±0.0002	ND	ND	ND
99	3-pentanol, 2-methyl-	1314	ND	ND	ND	0.4633±0.053	ND	0.3028±0.0263	ND	ND	ND
100	3-pyridinol	2381	ND	ND	ND	ND	0.017±0.0024	ND	ND	ND	ND
101	4-penten-2-ol, 4-methyl-	1125	ND	ND	ND	ND	0.0043±0.0005	0.0037±0.0004	ND	0.0005±0	ND
102	4-pyridinemethanol	2077	ND	ND	ND	ND	0.0125±0.0009	ND	ND	ND	ND
103	5-hexene-2-ol, 5-methyl-	1354	ND	ND	ND	ND	0.0019±0.0003	ND	ND	ND	ND
104	4-penten-2-ol, 3-methyl-	1125	0.0004±0.0001	0.0013±0.0001	ND	ND	ND	ND	ND	ND	ND
105	Benzyl alcohol	1852	0.0014±0.0002	0.002±0.0002	ND	0.003±0.0004	0.0056±0.001	0.0012±0.0001	0.0006±0.0004	0.0007±0.0001	0.0004±0.0002
106	Bicyclo[2.2.1]hept-5-en-2-ol	1514	ND	0.0004±0	ND	ND	0.0071±0.0007	0.0039±0.0003	ND	ND	ND
107	Bicyclo[2.2.2]octan-1-ol, 4-methyl-	1650	ND	ND	ND	ND	0.0058±0.0006	0.0043±0.0004	ND	ND	ND
108	Ethanol	405	ND	0.0106±0.0054	ND	0.0353±0.0121	0.043±0.0041	0.028±0.0033	ND	ND	ND
109	Cyclopropylmethanol	2060	ND	ND	ND	ND	ND	ND	ND	ND	0.0005±0.0002
110	DL-2,3-butanediol	1026	ND	ND	ND	ND	ND	ND	ND	0.0008±0	ND
111	Ethanol 2-(vinyloxy)-	2925	ND	ND	ND	ND	ND	ND	ND	0.1295±0.0126	ND

112	Ethanol 2,2'-oxydi-	2983	ND	0.0137±0.0014	ND	ND	ND	ND	ND	0.0182±0.0005	ND
113	Isopropanol	1047	ND	ND	ND	0.001±0.0004	0.0016±0.0002	ND	ND	ND	ND
114	Furfuryl alcohol, tetrahydro-5-methyl-, cis-	1437	ND	ND	0.0005±0.0001	ND	ND	ND	ND	ND	ND
115	Linalool	1531	0.0072±0.001	0.0055±0.0004	ND	0.0199±0.0022	0.0185±0.0024	0.0089±0.0028	0.0049±0.0011	0.0041±0.0005	0.0021±0.0002
116	Methyl mercaptan	621	0.0004±0.0002	0.0005±0.0001	ND	ND	ND	ND	0.0004±0.0001	ND	0.0003±0.0006
117	Phenethyl alcohol	1887	0.0045±0.0009	0.0068±0.0006	0.0017±0.0001	0.0114±0.0016	0.0212±0.0015	0.0147±0.0021	0.0012±0.0004	0.0020±0.0001	0.0014±0.0009
118	Isopentenol	1291	0.0205±0.0059	0.014±0.0009	0.0308±0.0037	0.0602±0.0062	0.0503±0.0061	0.0264±0.0038	0.0066±0.0015	0.0044±0.0003	0.0026±0.0005
119	Propylene glycol	1571	ND	0.0262±0.0039	0.0589±0.0060	0.0579±0.0063	0.0927±0.0092	ND	ND	ND	ND
120	Terpineol	1682	0.002±0.0003	0.0016±0.0001	ND	ND	ND	ND	0.0013±0.0003	0.0014±0.0002	ND
121	Trans-oxylinalool	1455	0.0152±0.0033	ND	ND	0.0472±0.0056	0.0751±0.0057	0.0507±0.0047	ND	0.0046±0.0005	0.0025±0.0003
122	Triethylene glycol	2722	ND	ND	0.0004±0.0001	ND	ND	ND	0.0006±0.0003	ND	ND
	**Phenols (15)**										
123	2-allylphenol	1706	0.0007±0.0001	0.002±0.0002	0.0007±0.0003	0.0018±0.0002	ND	0.0055±0.0005	ND	0.0023±0.0002	0.0019±0.0004
124	2-methoxy-4-vinylphenol	2168	0.0056±0.0018	0.0079±0.0008	ND	0.0188±0.0034	0.0292±0.0019	0.0199±0.0049	0.0033±0.0012	0.0028±0.0005	0.0038±0.0005
125	Isomalt	1597	0.0068±0.002	0.0059±0.0001	ND	0.0232±0.0023	0.0247±0.0032	0.0178±0.0033	ND	ND	ND
126	Maltol	1942	0.0225±0.007	0.0582±0.0011	ND	0.1071±0.018	0.1731±0.0396	0.1075±0.0057	ND	ND	ND
127	P-cresol	2052	0.0003±0	0.0007±0.0001	0.0002±0.0001	0.0008±0.0001	0.0022±0.0002	0.0015±0.0002	ND	ND	ND
128	phenol	2004	0.0212±0.0039	0.0624±0.0057	0.0213±0.0022	0.0583±0.0072	0.2006±0.016	0.1539±0.0142	0.0154±0.0033	0.055±0.0037	0.0507±0.0044
129	2,3-dimethylphenol	2118	ND	ND	ND	ND	ND	ND	ND	0.0005±0.0001	0.0034±0.0004
130	2,4-dimethylphenol	2052	ND	ND	ND	ND	ND	ND	ND	0.0005±0.0001	ND
131	2-ethylphenol	2042	ND	ND	ND	ND	ND	ND	ND	0.0003±0.0001	ND
132	Phenol 2-methoxy	1837	0.0328±0.0062	0.1101±0.0102	ND	0.0826±0.0098	ND	ND	0.0117±0.0025	0.0466±0.0063	0.03541±0.0043
133	2-methylphenol	1976	0.0022±0.0004	0.0065±0.0006	0.0018±0.0002	0.0057±0.0006	0.0205±0.0016	0.0135±0.0016	0.0021±0.0004	0.0078±0.001	ND
134	3-(1-methylethyl)-phenol	1301	ND	ND	ND	0.0007±0.0001	ND	ND	ND	ND	ND
135	3-methylphenol	2060	0.0013±0.0003	ND	ND	ND	ND	0.0054±0.0006	ND	ND	ND
136	4-ethylphenol	1167	0.002±0.0008	0.0047±0.0006	ND	0.0097±0.0009	0.0189±0.002	0.0142±0.0015	0.0003±0.0001	0.0033±0.0006	0.0012±0.0020
137	Phenol 4-ethyl-2-methoxy	2004	0.0032±0.0004	0.0097±0.001	0.0036±0.0001	0.0079±0.0012	0.0331±0.0022	0.0353±0.0063	0.0021±0.0004	0.0100±0.0015	0.0924±0.0113
	**Furan (38)**										
138	(2R, 5R)-2-methyl-5-(prop-1-en-2-yl) -2-vinyltetrahydrofuran	1160	0.0025±0.0008	0.0045±0.0004	ND	0.0111±0.0009	0.019±0.0016	0.0133±0.0011	0.0033±0.0004	0.0076±0.0007	ND

139	(2R, 5S)-2-methyl-5-(prop-1-en-2-yl) -2-vinyltetrahydrofuran	1203	ND	ND	ND	ND	0.0149±0.0015	ND	ND	0.0051±0.0004	0.0029±0.0002
140	2,2'-bisfuran	1588	0.0026±0.0006	0.0194±0.0014	0.0027±0.0003	0.0511±0.0065	0.0801±0.0056	0.0117±0.0006	0.011±0.0021	0.0194±0.0007	0.0443±0.0020
141	2,4-dimethylfuran	749	0.0013±0.0007	0.0042±0.0005	0.0023±0.0004	0.0043±0.0012	0.0142±0.0016	0.0196±0.0015	0.0017±0.0012	0.0034±0.0002	0.0072±0.0005
142	2-acetyl-5-methylfuran	1454	ND	ND	ND	0.4931±0.0611	0.2354±0.0204	0.5501±0.1324	0.0261±0.006	0.0433±0.0043	0.0268±0.0011
143	2-methoxytetrahydrofuran	767	0.0014±0.0005	0.0021±0.0001	ND	0.0035±0.001	0.0063±0.0006	0.0036±0.0001	ND	ND	ND
144	2-methyl-5-[(methylthio)methyl]furan	1531	ND	ND	ND	ND	ND	ND	0.0013±0.0003	ND	0.0023±0.0005
145	2-n-butylfuran	1044	ND	ND	ND	0.0014±0.0001	0.0383±0.0022	ND	ND	ND	ND
146	2-vinyl furan	929	ND	0.0942±0.01	0.0355±0.0030	0.1588±0.0332	0.305±0.0297	ND	ND	0.1164±0.0105	0.1092±0.0008
147	3-acetyl-2,5-dimethylfuran	1512	0.0038±0.0009	0.0051±0.0004	0.0082±0.0007	ND	ND	ND	ND	0.0041±0.0004	0.0016±0.0007
148	2,3-dihydrobenzofuran	1571	0.0053±0.001	0.0146±0.0013	0.0054±0.0005	0.0148±0.0017	0.0543±0.0047	0.045±0.0027	ND	0.0186±0.0016	0.0154±0.0002
149	2-methylbenzofuran	1576	0.0034±0.0006	0.0071±0.0005	0.0018±0.0001	0.0096±0.0012	0.028±0.0021	0.0183±0.0008	0.003±0.0002	0.0089±0.0008	0.0060±0.0003
150	4,7-dimethylbenzofuran	1688	ND	ND	ND	0.0012±0.0002	0.0062±0.0006	0.0036±0.0002	ND	ND	ND
151	Ethyl-2-benzofuran	1658	ND	0.0002±0	ND	ND	0.0008±0.0001	0.0012±0.0001	ND	ND	ND
152	Furan	748	0.0099±0.0058	0.034±0.0019	ND	ND	0.2526±0.028	0.1525±0.0094	0.0628±0.0108	0.0846±0.0082	0.0783±0.0063
153	Furan 2-(1,1-dimethylethyl)-4-methyl-	1980	ND	ND	ND	0.0011±0.0002	0.0195±0.0021	ND	ND	ND	ND
154	Furan 2-(2-furylmethyl)-5-methyl-	1659	0.01±0.0022	0.0347±0.003	0.0085±0.0027	0.0283±0.0033	ND	ND	0.0079±0.0014	0.039±0.0039	0.0307±0.0013
155	Furan 2-(methoxymethyl)-	1191	0.1009±0.0297	0.0008±0.0001	0.0010±0.0002	ND	ND	ND	0.0921±0.0162	0.1992±0.0117	0.1136±0.0019
156	Furan 2-(2-propenyl)-	970	ND	ND	ND	0.1041±0.0131	0.275±0.0236	0.1957±0.0094	0.0055±0.0024	0.0803±0.0243	0.0039±0.0005
157	Furan 2,2'-[oxybis(methylene)]bis-	1960	0.0166±0.0036	0.0308±0.0023	ND	0.0245±0.0026	0.0899±0.0053	0.0615±0.0028	ND	ND	ND
158	Furan, 2,2'-methylenebis[5-methyl-	1732	0.0008±0.0002	0.0022±0.0002	ND	ND	ND	ND	ND	0.0022±0.0002	0.0016±0.0001
159	Furan, 2,2'-methylenebis-	1616	ND	ND	ND	0.1727±0.0184	0.1565±0.0132	0.0151±0.0006	ND	ND	ND
160	2,3,5-trimethylfuran	881	ND	ND	ND	0.0071±0.0008	0.0143±0.0011	0.0051±0.0006	ND	0.0083±0.0005	0.0071±0.0011
161	2,3-dihydrofuran	234	0.0011±0.0001	0.0013±0.0001	ND	ND	ND	0.0025±0.0004	ND	ND	ND
162	Furan, 2,3-dihydro-5-methyl-	355	0.0086±0.0001	0.008±0.0008	ND	0.0158±0.0017	0.0213±0.0018	0.0137±0.0016	ND	ND	ND
163	Furan, 2,5-diethyltetrahydro-	852	ND	ND	ND	0.002±0.0002	0.0039±0.0004	0.0009±0.0001	ND	ND	ND
164	2,5-dimethylfuran	975	ND	0.0684±0.0123	0.0283±0.0028	0.1011±0.0077	0.2397±0.0325	0.1881±0.0186	ND	0.1091±0.0079	0.0985±0.0079
165	Furan 2-[[(methyldithio)methyl]-	1782	0.005±0.0011	0.0076±0.0007	0.0022±0.0001	0.0132±0.0016	0.0271±0.0019	0.0187±0.0019	0.0048±0.0009	0.0098±0.0009	0.0074±0.0003
166	Furan, 2-ethyl-5-methyl-	851	ND	ND	ND	0.0004±0	0.0449±0.0036	0.0374±0.0033	ND	0.0185±0.0018	0.0162±0.0018

167	Furan 2-[[(methylthio)methyl]-	1463	0.0678±0.0282	ND	0.0431±0.0268	ND	ND	ND	0.0374±0.0117	0.0471±0.0061	0.1810±0.0139
168	Furan, 2-methyl-	868	0.0772±0.0102	0.1958±0.0237	ND	0.2646±0.0247	0.5963±0.1279	0.5593±0.0537	ND	0.3734±0.0238	0.3503±0.0083
169	Furan, 2-methyl-5-(methylthio)-	1354	0.0017±0.0005	0.0039±0.0004	0.0013±0.0001	0.0041±0.0004	0.0158±0.0016	0.0125±0.0009	0.0014±0.0002	0.005±0.0004	0.0043±0.008
170	Furan, 2-pentyl	1184	0.0144±0.0031	0.02±0.0023	ND	0.0583±0.005	0.0842±0.0081	0.0663±0.0044	ND	0.0085±0.0008	0.0012±0.0014
171	Furan, 2-propyl-	811	ND	ND	ND	ND	ND	0.0115±0.0015	ND	0.0049±0.0005	ND
172	Furan, 3-phenyl	1833	ND	ND	ND	ND	ND	ND	0.0011±0.0002	0.002±0.0002	ND
173	Furan, tetrahydro-2-methyl-	1368	ND	0.0643±0.0069	0.0003±0.0001	0.0011±0.0005	0.0069±0.0004	0.0051±0.0003	ND	0.0012±0	0.0009±0.0001
174	Furan-2-carbonyl chloride, tetrahydro-	1330	ND	0.1679±0.0183	ND	0.0083±0.0019	0.6374±0.0815	0.041±0.003	ND	ND	ND
175	Tetrahydrofuran	869	0.0002±0.0001	ND	ND	0.0005±0	ND	ND	ND	ND	0.0002±0.0001
	**Sulfur-containing compounds (38)**										
176	2-acetyl-3-methylthiophene	1745	ND	ND	ND	0.0035±0.0005	0.0176±0.0012	0.0078±0.0006	ND	ND	0.0013±0.0006
177	2-acetyl-5-methylthiophene	1852	0.0004±0.0001	ND	0.0002±0.0000	0.0012±0.0002	ND	0.002±0.0002	ND	0.0005±0.0001	0.0004±0.0004
178	2-acetylthiazole	1627	0.0039±0.0009	0.004±0.0002	ND	0.011±0.0014	0.0153±0.0013	0.0101±0.0005	0.0007±0.0002	0.0013±0.0001	0.0004±0.0004
179	3(2H)-thiophene, dihydro-2-methyl-	1504	0.0608±0.0238	0.0527±0.0075	0.0298±0.0021	0.2265±0.0204	0.2362±0.0217	0.1263±0.0102	0.0231±0.0051	0.0108±0.0011	0.0133±0.010
180	3-acetyl-2,5-dimethylthiophene	1789	0.0022±0.0007	0.0025±0.0003	0.0058±0.0002	ND	ND	ND	ND	ND	ND
181	4-methylthiazole	1297	0.0204±0.0052	0.025±0.0021	ND	0.0505±0.0057	0.0956±0.01	0.061±0.0041	0.0142±0.003	0.0218±0.0015	0.0171±0.0023
182	5-eththiazole	1380	0.0016±0.0004	0.002±0.0002	ND	ND	0.0077±0.0009	0.0058±0.0004	0.0007±0.0002	0.0012±0.0002	0.0009±0.0005
183	Carbon disulfide	114	ND	ND	ND	ND	0.1139±0.0113	0.0583±0.0036	ND	0.0143±0.0018	0.0135±0.0011
184	Carbonyl sulfide	233	ND	ND	ND	ND	ND	ND	0.0007±0.0002	ND	ND
185	Cyclohexyl isothiocyanate	1651	ND	0.0029±0.0006	ND	ND	0.0071±0.0006	ND	ND	0.0021±0.0001	ND
186	Dihydro-2(3H)-thiophene	1620	0.0019±0.0003	0.0058±0.0004	ND	0.005±0.0004	0.0206±0.002	0.0207±0.0015	0.0005±0.0001	0.0024±0.0002	ND
187	Dihydro-3-(2H)-thiophene	1538	0.0159±0.0062	0.0133±0.0017	0.0377±0.0041	ND	0.057±0.0055	0.0348±0.0029	0.0039±0.0008	0.0016±0.0001	ND
188	Dimethyl sulfide	714	0.0018±0.0004	0.0031±0.0009	0.0054±0.0011	ND	0.0219±0.0024	0.0077±0.0016	0.0028±0.0005	ND	0.0010±0.0013
189	Dimethyl sulfoxide	1567	ND	ND	0.0033±0.0030	0.0504±0.0155	0.0482±0.0057	ND	ND	ND	ND
190	Dimethyl trisulfide	1351	0.0005±0.0001	0.0011±0.0001	ND	0.0021±0.0002	0.0053±0.0007	0.0015±0.0001	0.0053±0.001	0.017±0.0015	0.0070±0.004
191	Dimethyl disulfide	935	0.007±0.0033	0.0147±0.0013	ND	0.0244±0.0061	0.0548±0.0051	0.0275±0.0022	ND	ND	ND
192	Methyl ethyl disulfide	1058	ND	ND	ND	ND	ND	ND	ND	0.0016±0.0001	0.0015±0.0001
193	Thiazole	1208	0.0084±0.0024	0.0093±0.002	0.0027±0.003	0.0266±0.0039	0.0378±0.0039	0.0271±0.0024	0.0079±0.0018	0.0087±0.0014	0.0063±0.002
194	Thiazole, 2,4,5-trimethyl-	1357	0.0005±0.0002	0.0009±0.0002	0.0004±0.0001	0.0016±0.0002	0.0046±0.0005	0.0035±0.0002	ND	ND	ND

195	Thiazole, 2,4-dimethyl-	1293	0.0055±0.0019	0.0027±0.0002	ND	0.0172±0.0055	0.0454±0.0045	0.021±0.004	0.0013±0.0006	0.0047±0.0006	ND
196	Thiazole, 4,5-dimethyl-	1350	0.0016±0.0005	0.0034±0.0004	ND	ND	ND	ND	ND	ND	ND
197	Thiazole 2-ethyl-	1706	ND	ND	ND	0.0015±0.0002	0.0024±0.0004	0.002±0.004	ND	ND	ND
198	Thiazole 2-methyl-	1198	ND	ND	ND	0.0062±0.0052	0.0086±0.0012	0.0228±0.002	ND	0.0015±0.0001	ND
199	Thiazole, 4,5-dimethyl-	1350	ND	ND	ND	ND	ND	ND	ND	0.0041±0.0005	ND
200	Thiazole, 5-methyl-	1260	ND	ND	ND	0.0007±0.0001	0.0007±0.0001	ND	ND	ND	ND
201	Thiophene	765	0.0047±0.0026	0.01±0.0006	ND	0.0148±0.0051	0.0374±0.002	ND	0.0111±0.0077	0.0242±0.0019	ND
202	Thiophene 2-(1-methylethyl)-	1243	0.0006±0.0002	0.0068±0.0008	0.0014±0.0001	0.0056±0.0005	0.026±0.0028	0.0248±0.0018	ND	0.0012±0	ND
203	Thiophene 2,3,4-trimethyl-	1321	ND	0.0007±0.0001	ND	ND	0.0032±0.0004	ND	ND	ND	ND
204	2,5-dihydrothiophene	1003	ND	ND	0.0005±0.0001	ND	ND	ND	ND	ND	ND
205	Thiophene, 2,3-dihydro-5-methyl-	1222	ND	ND	ND	0.0021±0.0002	0.0133±0.0015	0.0082±0.0006	ND	ND	ND
206	2,3-dimethylthiophene	1158	ND	ND	ND	0.0013±0.0001	0.0044±0.0005	0.0048±0.0005	ND	0.0026±0.0004	0.0024±0.0001
207	2,4-dimethylthiophene	1131	ND	ND	ND	ND	ND	0.0098±0.0018	ND	ND	ND
208	2,5-dimethylthiophene	1092	ND	0.0011±0.0001	0.0005±0.0001	0.0017±0.0002	0.0038±0.0006	ND	ND	ND	ND
209	Thiophene, 2-vinyl-	1316	0.001±0.0003	0.0029±0.0002	0.0095±0.0004	0.0046±0.0005	0.0093±0.0012	0.0084±0.0006	ND	0.0034±0.0002	0.0043±0.0010
210	Thiophene 2-methyl	1023	0.0019±0.0009	ND	ND	0.0061±0.001	0.0008±0.0001	0.0009±0.0001	ND	0.0649±0.0047	0.0538±0.0023
211	Thiophene 3,4-diethyl-	1394	ND	0.0014±0.0001	0.0053±0.0007	ND	0.0054±0.0006	0.0045±0.0003	ND	0.0018±0.0001	ND
212	Thiophene 3-ethyl	1148	ND	ND	ND	ND	ND	ND	ND	0.0014±0	0.0013±0.0001
213	Thiophene 3-methyl	1023	ND	0.0044±0.001	ND	0.0354±0.0062	0.0097±0.0014	ND	ND	0.0159±0.0009	ND

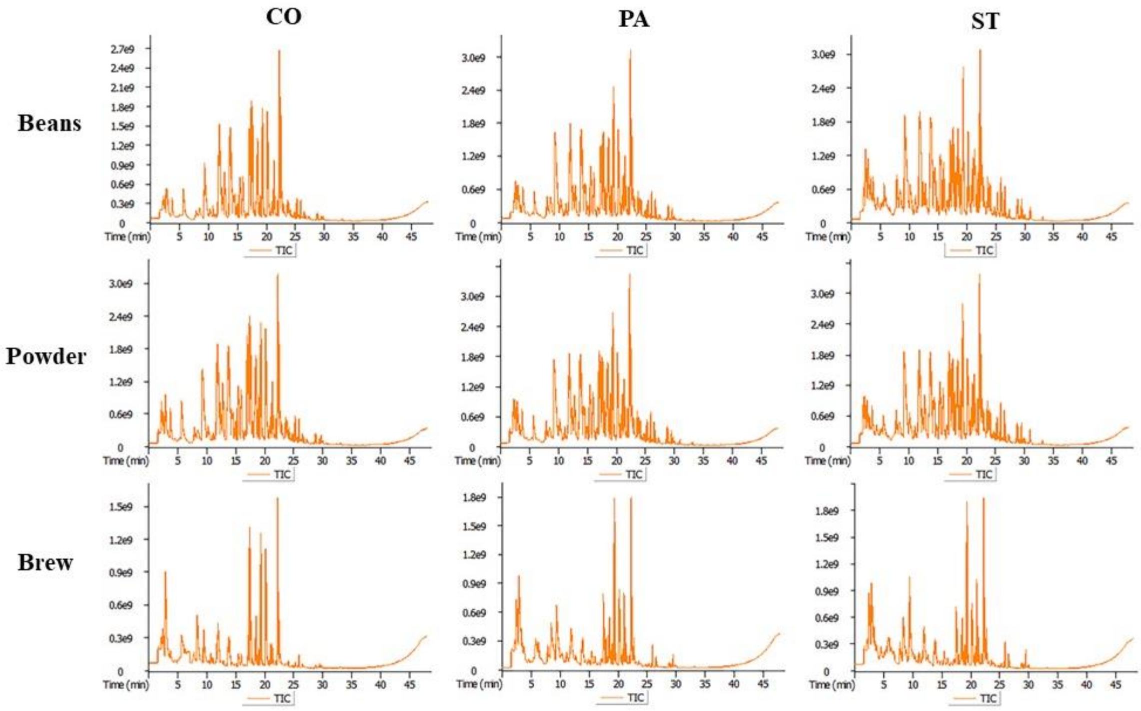

Figure S1. Total Ion flow chromatograms of three brands of coffee (CO, PA, ST) under different states states (beans, powder, and brews) from GC-TOF/MS

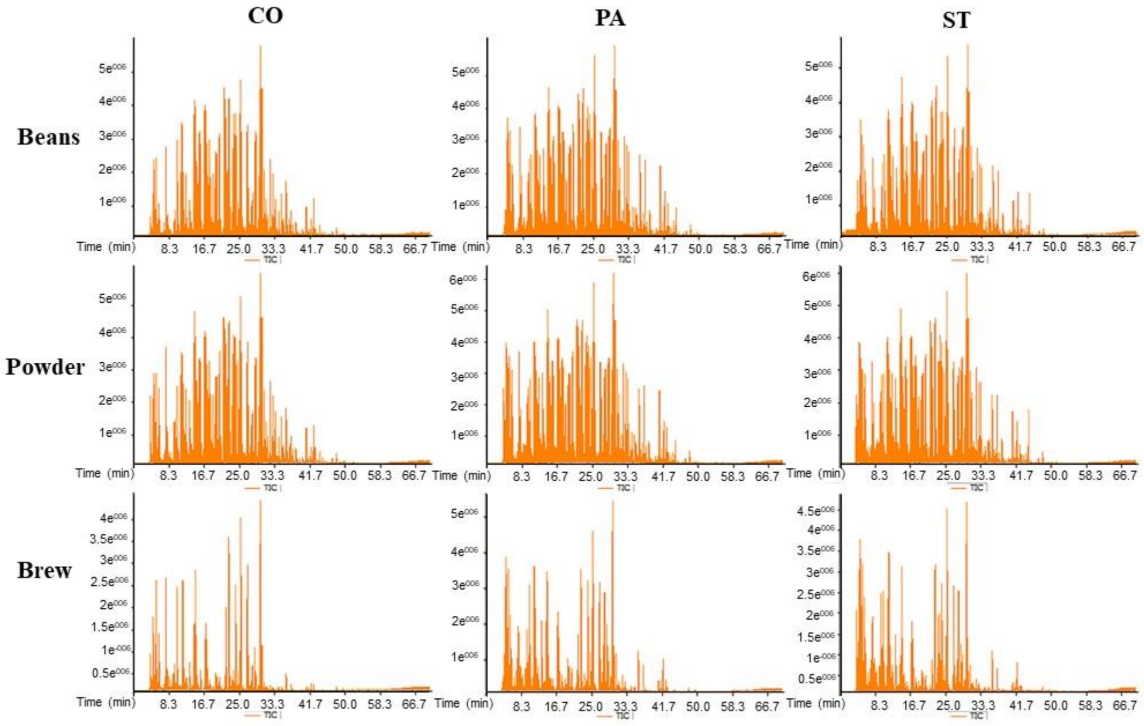

Figure S2. Total Ion flow chromatograms of three brands of coffee (CO, PA, ST) under different states states (beans, powder, and brews) from GC×GC-TOF/MS on the first column
